# The serodominant secreted effector protein of *Salmonella*, SseB, is a strong CD4 antigen containing an immunodominant epitope presented by diverse HLA class II alleles

**DOI:** 10.1111/imm.12327

**Published:** 2014-10-02

**Authors:** Catherine J Reynolds, Claire Jones, Christoph J Blohmke, Thomas C Darton, Amelie Goudet, Ruhena Sergeant, Bernard Maillere, Andrew J Pollard, Daniel M Altmann, Rosemary J Boyton

**Affiliations:** 1 Section of Infectious Diseases and Immunity, Department of Medicine, Imperial College, Hammersmith HospitalLondon, UK; 2 Oxford Vaccine Group, Department of Paediatrics, NIHR Oxford Biomedical Research Centre, University of OxfordOxford, UK; 3 CEA-Saclay, Institute of Biology and TechnologiesGif Sur Yvette, France; 4 Imperial College Healthcare NHS Trust, Hammersmith HospitalLondon, UK

**Keywords:** CD4 epitope, HLA-DR transgenic, *Salmonella*, SseB, type 3 secretion system

## Abstract

Detailed characterization of the protective T-cell response in salmonellosis is a pressing unmet need in light of the global burden of human *Salmonella* infections and the likely contribution of CD4 T cells to immunity against this intracellular infection. In previous studies screening patient sera against antigen arrays, SseB was noteworthy as a serodominant target of adaptive immunity, inducing significantly raised antibody responses in HIV-seronegative compared with seropositive patients. SseB is a secreted protein, part of the Espa superfamily, localized to the bacterial surface and forming part of the translocon of the type III secretion system (T3SS) encoded by *Salmonella* pathogenicity island 2. We demonstrate here that SseB is also a target of CD4 T-cell immunity, generating a substantial response after experimental infection in human volunteers, with around 0·1% of the peripheral repertoire responding to it. HLA-DR/peptide binding studies indicate that this protein encompasses a number of peptides with ability to bind to several different HLA-DR alleles. Of these, peptide 11 (p11) was shown in priming of both HLA-DR1 and HLA-DR4 transgenic mice to contain an immunodominant CD4 epitope. Analysis of responses in human donors showed immunity focused on p11 and another epitope in peptide 2. The high frequency of SseB-reactive CD4 T cells and the broad applicability to diverse HLA genotypes coupled with previous observations of serodominance and protective vaccination in mouse challenge experiments, make SseB a plausible candidate for next-generation *Salmonella* vaccines.

## Introduction

There are estimated to be > 21 million cases of typhoid fever annually. *Salmonella* infections cause outbreaks of gastroenteritis in industrialized nations and enteric fever and non-typhoidal *Salmonella* bacteraemia in resource-poor regions of the world, killing over 600 000 people annually.^1^ Disseminated non-typhoidal *Salmonella* infections are strongly associated with HIV infection and young age in Asia and Africa.^2^,^3^ Since infection is food- and water-borne, *Salmonella* infection is endemic in regions where safe drinking water supply and adequate sewage disposal are unavailable.

There are two widely licensed vaccines for typhoid, but they offer only moderate protection and are not widely used in endemic areas.^4^ The Vi polysaccharide vaccine targets antibody to the capsule of *Salmonella* Typhi, in a T-cell-independent response and therefore lacks immunogenicity in children under 2 years old. The live oral vaccine, Ty21a, requires three or four oral doses for moderate efficacy and is not licensed for children under 6 years of age. Protein–polysaccharide conjugate vaccines for typhoid, again targeting antibody to the Vi capsule, have recently been licensed in India and other conjugated and live-attenuated vaccines are in development,^5^,^6^ but there are no vaccines yet available to prevent paratyphoid or non-typhoidal *Salmonella* infection There is a considerable challenge posed by the need to generate broad serovar coverage in a form that would be suitable and effective for use in all susceptible individuals, including young infants, and would offer lasting immune memory.

Many facets in the immune control of salmonellosis remain poorly understood. There is evidence of the need for T-cell and antibody-mediated control.^7^ In the case of *S.* Typhi infection, up to 20% of infected individuals may have recurring infection until a protective state is eventually attained, or they go on to relapse.^8^,^9^ There is only a partial picture of the nature of CD4 T-cell immunity during *Salmonella* infections and the identity of the key *Salmonella* T-cell antigens is unknown. Different phases of host–pathogen interaction in the murine model of *Salmonella* infection have been described.^7^ In the initial phase, bacteria enter tissues, normally via the gut, then disseminate via the blood to the bone marrow, liver, spleen and other tissues. Rapid bacterial growth is countered by innate cytotoxicity, mainly through reactive oxygen intermediates as well as antibody/complement-mediated clearance. Once bacteria start to replicate intracellularly, innate immunity remains important, for example, through Nramp-1, as well as the linked activation of adaptive immunity.^7^ Interferon-*γ* (IFN-*γ*) is probably derived largely from natural killer cell activation initially, and subsequently from T helper type 1 (Th1) cells,^10^ and the importance of this pathway in defence is shown by the preponderance of *Salmonella* infections among individuals with interleukin-12/IFN-*γ* defects.^11^ During the final phase, bacterial levels in tissues fall until clearance is achieved. Adaptive T-cell immunity is seemingly not required for initial control of infection as mice deficient in CD4 cells, CD8 cells or both can control initial bacterial growth.^12^ T-cell-mediated immunity is impaired in the absence of B cells.^13^ Specific T-cell cytokine programmes have not been exhaustively mapped, but current evidence suggests an important role for Th1 immunity. McSorley's group used tetramers loaded with epitopes from *Salmonella* flagellin or from SseJ to track the phenotype of responding CD4 T cells during murine challenge.^14^ In line with the notion of Th1 predominance, Tbet was the key CD4 transcription factor induced, though there was skewing to Th17 responses among flagellin-specific, gut CD4 T cells.^14^ Despite this being an intracellular pathogen, mice lacking CD8 cells show delayed bacterial clearance rather than substantially enhanced disease susceptibility.^15^

There have been a limited number of studies aimed at exploring candidates from the *S*. Typhi immunome for serodiagnostic, serodominant or protective credentials with respect to B-cell or T-cell immunity.^16^–^19^ Some candidate antigens have been successfully deployed to induce protective immunity against challenge in mice; these antigens include OmpL, SseB, SseD and CirA.^16^,^18^,^19^ Antigen arrays have been informative: an array of 2724 *Salmonella enterica* serovar Typhi antigens, selected on the basis of key terms such as ‘outer membrane’ or ‘chaperone’, representing over 63% of the proteome was screened for responses in immune sera from mice infected with *Salmonella* Typhimurium BRD509.^18^ A total of 117 antigens were detected, some of these differentially between the different resistant or susceptible mouse strains used. Studies with patient sera identified 16 seroreactive antigens differentially bound by sera from patients with acute typhoid and healthy controls.^18^ Fourteen of these antigens were detected in sera from Malawian paediatric acute cases and SseB was noteworthy as a target of adaptive immunity, inducing significantly raised antibody responses in HIV-seronegative compared with HIV-seropositive subjects. In studies on protection of C57BL/6 mice from oral challenge with *Salmonella* SL1344, immunization with two doses of SseB offered substantial protection with respect to mean survival time and tissue bacterial burden.^18^

SseB is a secreted protein, part of the Espa superfamily, localized to the bacterial surface and forming part of the translocon of the type III secretion system (T3SS) encoded by the *Salmonella* pathogenicity island 2 (Spi2).^19^ The other components include SseC and SseD. The T3SS of *Salmonella* is induced during the intracellular phase of infection when bacteria are found in the *Salmonella*-containing vacuole and must be able to translocate effector proteins across the host cell plasma membrane.^20^,^21^ The role of SseB is to promote membrane pore formation, allowing proteins to access host cytoplasm: SseB mutant strains lack the ability to translocate SPI2 effector proteins during intracellular infection of macrophages.^22^

The EspA protein of enteropathogenic *Escherichia coli* (EPEC), which is an orthologue of *Salmonella* SseB, has been evaluated as a protective vaccine candidate in EPEC.^23^ In seeking to further establish the credentials of SseB as a *Salmonella* vaccine candidate antigen, we here set out to validate the credentials of SseB as a target of CD4 immunity in the context of *Salmonella* infection and determine the impact of HLA class II polymorphisms on immune recognition of immunodominant epitopes.

## Materials and methods

### 

#### SseB protein and peptide panel

Recombinant SseB protein was produced and purified after codon optimization and sub-cloning into the vector pBMH for expression in *E. coli* (Biomatik, Wilmington, DE). An SseB peptide library comprising 20-mers overlapping by 10 amino acids was synthesized by GL Biochem (Shanghai) Ltd (Shanghai, China; Table1).

**Table 1:** Amino acid sequences of 18 overlapping SseB peptides used for HLA binding, HLA transgenic and human epitope mapping studies. Peptides are 20 amino acids in length and adjacent peptides overlap by 10 amino acids

Peptide	Peptide name	Amino acid sequence
1	T1266 [1–20]	MSSGNILWGSQNPIVFKNSF
2	T1266 [11–30]	QNPIVFKNSFGVSNADTGSQ
3	T1266 [21–40]	GVSNADTGSQDDLSQQNPFA
4	T1266 [31–50]	DDLSQQNPFAEGYGVLLILL
5	T1266 [41–60]	EGYGVLLILLMVIQAIANNK
6	T1266 [51–70]	MVIQAIANNKFIEVQKNAER
7	T1266 [61–80]	FIEVQKNAERARNTQEKSNE
8	T1266 [71–90]	ARNTQEKSNEMDEVIAKAAK
9	T1266 [81–100]	MDEVIAKAAKGDAKTKEEVP
10	T1266 [91–110]	GDAKTKEEVPEDVIKYMRDN
11	T1266 [101–120]	EDVIKYMRDNGILIDGMTID
12	T1266 [111–130]	GILIDGMTIDDYMAKYGDHG
13	T1266 [121–140]	DYMAKYGDHGKLDKGGLQAI
14	T1266 [131–150]	KLDKGGLQAIKAALDNDANR
15	T1266 [141–160]	KAALDNDANRNTDLMSQGQI
16	T1266 [151–170]	NTDLMSQGQITIQKMSQELN
17	T1266 [161–180]	TIQKMSQELNAVLTQLTGLI
18	T1266 [171–196]	AVLTQLTGLISKWGEISSMIAQKTYS

#### HLA-transgenic mice

Mice transgenic for HLA-DR1 (that is, HLA-DRA1*0101/HLA-DRB1*0101) and HLA-DR4 (that is, HLA-DRA1*0101/HLA-DRB1*0401)^24^–^26^ were bred and housed in the Centre for Biological Services at the Hammersmith campus, Imperial College London, UK, in accordance with Home Office regulations and College ethics review. Both transgenic lines are maintained on a C57BL/6 background as homozygotes for knockout of murine MHC Class II H2-A*β*.^27^

#### Human peripheral blood mononuclear cells donors

CD4 T cell responses to SseB were analysed in peripheral blood mononuclear cell (PBMC) samples from healthy control donors with no reported clinical history of *Salmonella* infection and from donors who had been experimentally challenged with *S*. Typhi 12 months previously as described elsewhere.^28^ Healthy control blood samples were collected with informed consent, under the approval of Ethics REC reference number 08/H0707/173. The *S*. Typhi challenge participants were recruited by the Oxford Vaccine Group, University of Oxford and the study was performed at the Centre for Clinical Vaccinology and Tropical Medicine, Oxford. Ethics approval was obtained from the NRES Committee South Central – Oxford A (11/SC/0302). Written, informed consent was provided by all participants. A pre-determined bacterial dose (10^4^ colony-forming units) of challenge agent (Quailes strain) was delivered by oral ingestion suspended in a sodium bicarbonate solution.^28^ Study exclusion criteria included previous typhoid vaccination, residence in a typhoid endemic country for over 6 months and known diagnosis of a probably or confirmed typhoid infection. Blood samples were collected and PBMCs were purified by Lymphoprep density gradient separation.

#### Human IFN-*γ* ELISpot

Human IFN-*γ* ELISpot assays were run using reagents from Mabtech (Stockholm, Sweden). Briefly, 2 × 10^5^ thawed PBMCs were incubated in pre-coated human IFN-*γ* ELISpot plates, in triplicate, with 50 μg/ml of SseB peptide or recombinant protein (25 μg/ml) in RPMI-1640 medium containing 2 mm l-glutamine, 50 U/ml penicillin G, 50 μg/ml streptomycin sulphate and 10% fetal calf serum. Cells were cultured at 37° and in 5% CO_2_ for 24 hr before assay development using an horseradish peroxidase-labelled detection antibody and Tetramethylbenzidine (TMB) substrate. Developed plates were left to dry before spot counting using an AID ELISpot reader (Autoimmun Diagnostika GMBH, Straßberg, Germany). Positive epitopes for a given individual were defined as a greater number of spot-forming cells than 2 SD over the mean of the no protein/peptide control triplicate. To better define HLA restriction, human donor ELISpot responses were also analysed in the presence of HLA class II blocking monoclonal antibodies at a final concentration of 25 μg/ml: L243 (anti-HLA-DR*α*), SPVL-3 (anti-HLA-DQ), B7/21 (anti-HLA-DP) and control mouse IgG.

#### Murine IFN-*γ* ELISpot

HLA-DR1 or HLA-DR4 transgenic mice were primed, by subcutaneous injection into the footpad, with 25 μg of recombinant SseB protein administered as a 1 : 1 emulsion with TiterMax® Gold (CytRx Corporation, Los Angeles, CA). Ten days after priming, popliteal draining lymph nodes were harvested and stimulated, in triplicate, at 2 × 10^5^ cells per well with 25 μg of SseB peptide or recombinant protein in pre-coated murine IFN-*γ* ELISpot plates (2BScientific, Upper Heyford, Oxfordshire, UK). Culture medium was RPMI-1640 containing 2 mm l-glutamine, 50 U/ml penicillin G, 50 μg/ml streptomycin sulphate, 50 μm β-mercaptoethanol and 10% fetal calf serum. Cells were cultured at 37° and in 5% CO_2_ for 72 hr before assay development using a biotinylated anti-IFN-*γ* antibody, a horseradish peroxidase detection antibody and TMB substrate (2BScientific). Developed plates were left to dry before spot counting using an AID ELISpot reader (Autoimmun Diagnostika GMBH). Positive epitopes for a given transgenic line were defined as those where more than half of assayed animals gave a greater number of spot-forming cells than 2 SD over the mean of the no protein/peptide control.

#### Peptide/HLA-DR binding assays

HLA-DR heterodimers were purified from homozygous Epstein–Barr virus B-lymphoblastoid cell lines (HPA Culture Collections, Salisbury, UK) by affinity chromatography with the monomorphic HLA-DR*α* monoclonal antibody, L243.^29^,^30^ Binding of the SseB peptide panel to the HLA-DR molecules HLA-DRB1*01:01, 03:01, 04:01, 07:01, 09:01, 11:01, 12:02, 15:01, 15:02 was assessed by competitive ELISA, as previously described, using an automated workstation.^29^,^30^ We evaluated the peptide concentration able to prevent binding of 50% of the labelled peptide (IC_50_). Data were expressed as relative affinity: ratio of the IC_50_ of the peptide to the IC_50_ of the reference peptide, which is a high binder to the HLA class II molecule. Unlabelled forms of the biotinylated peptides were used as reference peptides. Their sequences and IC_50_ values were the following: HA 306–318 (PKYVKQNTLKLAT) for DRB1*01:01 (2 nm), DRB1*04:01 (69 nm), DRB1*07:01 (30 nm) and DRB1*1101 (36 nm), MT2-16 (AAKTIAYDEEARRGLE) for DRB1*03 :01 (84 nm), CTP 427–441 (VHGFYNPAVSRIVEA) for DRB1*0901 (15 nm), TFR 141–155 (TGTIKLLNENSYVPR; 73 nm) for DRB1*1202, A3 152–166 (EAEQLRAYLDGTGVE) for DRB1*15:01 (37 nm) and TFR 607–620 (LNLDYERYNSQLLS) for DRB1*15:02 (3 nm).

## Results

### The SseB sequence encompasses peptides with broad binding to multiple HLA-DR alleles

The HLA-DR peptide-binding assay used here was designed to give a strong coverage of commonly expressed alleles across diverse populations. For example, the DR1, 3, 4, 7, 11 and 1501 heterodimers cumulatively cover approximately 80% of individuals in Caucasian populations, whereas DR9, DR1202 and DR1502 are rare in Caucasian populations but are found in around a third of individuals in many South East Asian populations. Of the 18 peptides encompassing the SseB overlapping peptide library, 15 show significant HLA-DR binding (Table2). Eight peptides were able to bind specifically to only one or two alleles, but the seven other binders were able to bind more heterodimers. It is noteworthy that there are three peptides, p2, p11 and p18, with the property of binding 6, 7 and 8 different HLA-DR heterodimers, respectively. They span HLA-DRB sequences with rather diverse peptide-binding pockets.

**Table 2:** Relative binding of SseB peptides to HLA-DR heterodimers

Peptide	CD4 T-cell epitope	Relative binding affinity of peptide to HLA-DR molecules^1^								

		DRB1	DRB1	DRB1	DRB1	DRB1	DRB1	DRB1	DRB1	DRB1
		*0101	*0301	*0401	*0701	*0901	*1101	*1202	*1501	*1502
1	^1^MSSGNILWGSQNPIVFKNSF^20^	447	> 119	**12**	**6**	> 689	> 278	**3**	**7**	> 3 333
2	^11^QNPIVFKNSFGVSNADTGSQ^30^	300	> 119	**9**	**14**	**10**	> 278	**59**	**0·4**	**5**
3	^21^GVSNADTGSQDDLSQQNPFA^40^	> 4149	> 119	> 145	> 336	> 689	ND	> 136	> 270	> 3 333
4	^31^DDLSQQNPFAEGYGVLLILL^50^	**22**	> 119	> 145	**6**	**28**	ND	> 136	**10**	**55**
5	^41^EGYGVLLILLMVIQAIANNK^60^	> 4149	> 119	> 145	> 336	> 689	> 278	> 136	> 270	> 3 333
6	^51^MVIQAIANNKFIEVQKNAER^70^	235	> 119	**6**	**2**	> 689	**8**	**2**	**1**	577
7	^61^FIEVQKNAERARNTQEKSNE^80^	4500	**89**	109	> 336	> 689	> 278	> 136	211	> 3 333
8	^71^ARNTQEKSNEMDEVIAKAAK^90^	> 4149	> 119	> 145	> 336	> 689	> 278	> 136	> 270	> 3 333
9	^81^MDEVIAKAAKGDAKTKEEVP^100^	1508	**41**	> 145	> 336	626	**8**	> 136	200	> 3 333
10	^91^GDAKTKEEVPEDVIKYMRDN^110^	> 4149	> 119	> 145	**87**	> 689	> 278	> 136	**58**	> 3 333
11	^101^EDVIKYMRDNGILIDGMTID^120^	**61**	> 119	**17**	**2**	**34**	> 278	**8**	**0·3**	**37**
12	^111^GILIDGMTIDDYMAKYGDHG^130^	> 4149	**18**	> 145	167	> 689	> 278	105	**12**	1 826
13	^121^DYMAKYGDHGKLDKGGLQAI^140^	2958	> 119	> 145	> 336	> 689	> 278	> 136	**12**	2 000
14	^131^KLDKGGLQAIKAALDNDANR^150^	335	> 119	**13**	**17**	**50**	> 278	**71**	**10**	> 3 333
15	^141^KAALDNDANRNTDLMSQGQI^160^	> 4149	**57**	> 145	> 336	> 689	> 278	> 136	> 270	> 3 333
16	^151^NTDLMSQGQITIQKMSQELN^170^	964	> 119	> 145	> 336	> 689	> 278	**52**	> 270	> 3 333
17	^161^TIQKMSQELNAVLTQLTGLI^180^	**18**	> 119	> 145	**8**	> 689	> 278	> 136	> 270	> 3 333
18	^171^AVLTQLTGLISKWGEISSMIAQKTYS^196^	**2**	> 119	**2**	**12**	**21**	**7**	**4**	**0·5**	**63**

1 Results are expressed as a relative binding ratio obtained by dividing the IC_50_ of peptide by that of a reference peptide that binds strongly to the HLA molecule. Lower numbers correspond to a higher binding affinity. Numbers in bold with a ratio of 20 or less = high affinity binding; number in bold with a ratio of 20–100 = moderate affinity binding. Each peptide–MHC combination was evaluated in three independent experiments.

### Mapping immunodominant SseB epitopes in HLA-DR transgenic mice

**Figure 1:**
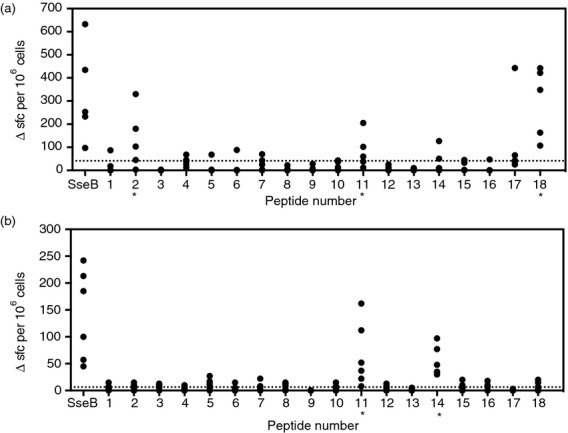
Definition of HLA-DR specific T-cell epitopes in DR1 and DR4 transgenic mice. (a) HLA-DR1 (*n* = 5) and (b) HLA-DR4 (*n* = 6) mice were footpad primed with ssEB protein in combination with TiterMax® Gold adjuvant. Draining lymph node (DLN) cells were assayed by ELISpot at day 10 for interferon-*γ* (IFN-*γ*) T-cell responses to ssEB peptides and protein. A peptide was defined as containing a T-cell epitope for DR1 or DR4 HLA molecules if more than half of the mice assayed gave a greater number of spot-forming cells than 2 SD over the mean of the no peptide/protein control. The 2 SD value for each transgenic line is shown by a dotted line.

There are clear logistical problems inherent in the elucidation of patterns of HLA class II restriction in outbred human populations: a CD4 T-cell response to a given peptide may result from presentation through any one of the several different class II heterodimer species expressed on each antigen-presenting cell. We have therefore favoured the approach of complementing analysis of human donor responses with analysis in immunized HLA transgenics.^24^–^26^,^30^ HLA-DR1 and HLA-DR4 transgenic lines, each also carrying a homozygous deletion for endogenous murine H2-A*β*, were immunized with recombinant SseB protein (Fig.1a,b). Ten days later, recall responses were tested by IFN-*γ* ELIspot of popliteal lymph node T cells. Immunization of HLA-DR1 transgenics shows that whole SseB protein is highly immunogenic in this strain and that p2, p11 and p18 peptides carry HLA-DR1 restricted immunodominant CD4 epitopes (Fig.1a). In HLA-DR4 transgenics, there is again a relatively large response to the whole SseB protein, with CD4 epitopes detected in p11 and p14 (Fig.1b). Hence, p11, which binds with moderate to high affinity to the seven HLA-DR heterodimers screened, is shown to be functionally relevant to the HLA-DR1 and -DR4 CD4 T-cell repertoire. Peptide 18, which can bind to both DR1 and DR4, was found to contain a functional CD4 epitope in the context of DR1 but not DR4.

### Human CD4 T-cell epitopes from SseB

**Figure 2:**
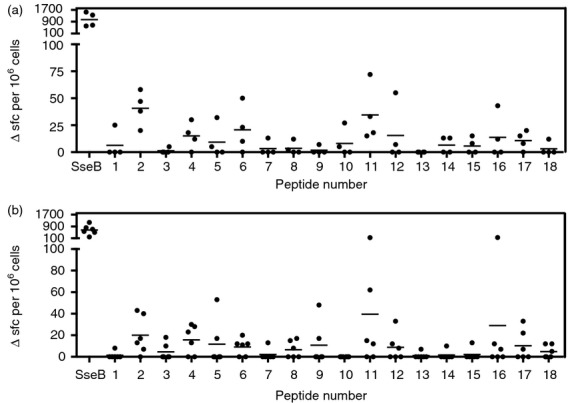
T-cell responses to peptides of ssEB, particularly peptides 2 and 11, are detectable in peripheral blood mononuclear (PBMC) samples from *Salmonella*-exposed individuals. Peripheral blood samples (PBMC) (a) *Salmonella* exposed (*n* = 4) and (b) healthy control (*n* = 6) individuals were assayed by ELISpot for IFN-*γ* T-cell responses to SseB peptides and protein. Response of a given individual to a particular peptide was defined as positive if the number of spot-forming cells was > 2 SD over the mean of the no peptide/protein control for that individual. Responses not defined as positive are plotted as zero. Mean spot-forming cells per 10^6^ PBMC for each group is represented by a straight line.

We then analysed human CD4 T-cell IFN-*γ* ELIspot responses to SseB and the SseB peptide library. Two human cohorts were used, one comprising donors who had 12 months previously been challenged with 10^4^ colony-forming units of live *S*. Typhi strain and the other comprising healthy volunteers with no known specific clinical history of *Salmonella*-related illness (Fig.2a,b). Response of a given individual to a particular peptide from the SseB library was defined as positive if the number of spot-forming cells was > 2 SD above the mean of the no-peptide/protein control for that individual. Particularly noteworthy in this analysis is that cells from both cohorts show a very sizeable T-cell response to whole SseB protein, approaching 1 in a 1000 of the peripheral repertoire; this suggests that the adaptive immune system was significantly exposed to *Salmonella* SseB (or its cross-reactive orthologues in related pathogens) and devotes a sizeable immune repertoire to its recognition. All of the individuals screened across the two cohorts respond to whole SseB and to at least some of the peptides. Across both cohorts, the largest and most common peptide responses are to p2 and p11, the key epitope predicted by the HLA transgenic mouse screen. However, p18, which had been identified as a peptide with broad HLA-DR binding and as an epitope in HLA-DR1 transgenics but not in HLA-DR4 transgenics, is less frequent among the epitopes recognized by the human donor cells. Two of the four challenged donors became clinically unwell with typhoid and two did not; the former pair were characterized by a narrower CD4 response focused on p2 and p11, while the latter pair mounted a response to a larger number of epitopes.

All of the donors for this study were HLA genotyped (Table3), but no particular HLA class II alleles were identified that correlated with patterns of response, including p2 and p11 recognition. Attempts were made to use HLA class II monoclonal antibody blocking studies of bulk responses for imputing dominant HLA restriction of responses; due to consequences of cross-linking between class II molecules, these studies can, in our experience, as often show enhancement of functional responses as blocking. We nevertheless undertook extensive additional studies of this type in an attempt to illuminate the nature of p2 and p11. While anti-DR blocking studies (with L243) indeed tended to show enhancement of responses (data not shown), we identified clear and specific inhibition by the anti-DQ monoclonal antibody, SPVL-3 in the p11 response of donor EC02 (see Supporting information, Fig. S1). This suggests that the very broad responses to these immunodominant epitopes may result in part from the ability to be presented both by HLA-DR and HLA-DQ isotypes.

**Table 3:** HLA class II genotypes of donors for this study

Subject	HLA class II			

	DRB1		DQB1	
HC01	1	103	5	5
HC02	1	13	5	6
HC03	1	17	2	5
HC04	13	13	6	7
EC01	15	7	2	6
EC02	1	15	5	6
EC03	4	13	6	8
EC04	17	4	2	8

### Variation in the SseB p11 epitope across *Salmonella enterica* serovars

**Figure 3:**
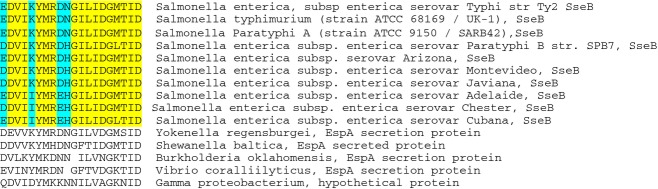
Conservation of the p11 T-cell epitope across *Salmonella* and other bacterial species. Conserved residues across *Salmonella* clades are shown in yellow, variant residues in turquoise.

In light of the immunoprevalence of the SseB p11 epitope as determined across HLA polymorphisms in both transgenic mice and in humans, it was important to determine the extent to which this epitope might vary across enterica serovars (Fig.3). Positions 1, 5, 9 and 10 within the 20-mer p11 are variable across *S. enterica* strains, possibly arguing for selection driven by host T-cell immunity. The epitope is to a degree conserved in more distantly related species than members of the Espa superfamily, such as *Yokenella* and *Vibrio* sp.

## Discussion

There has been relatively little functional and immunogenetic dissection of CD4 T-cell immunity to *Salmonella* in the context of human infection. Studies on human live-attenuated vaccine volunteers at the Center for Vaccine Development (University of Maryland) show that specific responses encompass both IFN-*γ* and interleukin-17 from polyfunctional T cells, with CD4 and CD8 responses both induced.^31^ Sheikh *et al*.^32^ used *S*. Typhi membrane preparation proteins to analyse the IFN-*γ* ELIspot responses of acute or convalescent typhoid patients in Bangladesh. Seven different antigens were studied, strong CD4 responses being observed in acute and convalescent patient samples in response to StaF, CsgF, PagC, StbB, CdgD, OppA and STY2195. A recent study characterized CD4 and CD8 responses in C57BL/6 mice following oral challenge with live *S*. Typhimurium.^33^ Predictive algorithms were used to select some 1400 predicted antigenic epitopes, conserved across Typhi and Typhimurium sequences, yielding clear responses to the alkyl hydroperoxide reductase subunit C (AhpC), the ethanolamine ammonia-lyase small subunit (EutC) and in STM1540, a putative hydrolase. The AhpC epitope was of particular interest, being highly conserved across several other pathogens including *Pseudomonas*, *Burkholderia* and *Citrobacter* sp. However, prophylactic vaccination with the immunodominant CD4 epitopes did not significantly enhance bacterial clearance.^33^

Serodominant antigens picked up as targets through screening of patient sera often contain strong CD4 T-cell epitopes. We here investigated whether, SseB, a component of the *Salmonella* T3SS and found to be strongly seroreactive in analysis of both infected mice and a Malawian paediatric cohort^18^ was strongly recognized by CD4 T cells as well. A key finding is that there is a very high frequency of SseB-specific CD4 T cells, whether in immunized HLA-DR transgenics, donors who have been through a live *S*. Typhi challenge protocol or indeed in healthy controls. The very substantial response in the human donors, around 0·1% of the repertoire, argues either that individuals can be strongly primed asymptomatically through repeated exposure to food-borne *Salmonella*, or that, because SseB is a member of the relatively conserved Espa superfamily of bacterial T3SS proteins, there is a degree of ongoing, cross-reactive immune stimulation through epitopes from, for example, *Vibrio* species (Figure3). We found amino acid residues 1, 5, 9 and 10 within p11 to be variable across *S. enterica* strains, which may be compatible with the notion of selection driven by adaptive immunity, although the sequence is conserved across typhoid and paratyphoid sequences. While our binding studies show p11 to be a rather promiscuous binder, able to bind with moderate or high affinity to several HLA-DR alleles, predictive algorithms such as NetMHCII suggest that this range of binding would not extend also to presentation by HLA-DQ heterodimers. NetMHCII predicts a core binding sequence of YMRDNGIL for HLA-DR1 and of IKYMRDNGILI for HLA-DR15. Hence, the variability between *S. enterica* serovars at amino acid 1 of p11 where there is an E/D switch between variants is likely to be irrelevant to HLA class II binding and T-cell receptor recognition. The tyrosine is predicted, for HLA-DR alleles including DR1, DR4, DR9 and DR1502, to sit in pocket 1 with the variable, albeit conservative, D/E in pocket 4, and so probably to impact on differential interactions with HLA-DR heterodimers. The N/H dimorphism at P5 is predicted to lie at the key T-cell receptor contact residue, and so the difference is highly likely to impact on T-cell activation. A second binding register seems to be shared by HLA-DR7 and HLA-DR1501 and uses the I in position 4 as a P1 anchor residue. D/E and H/N dimorphisms affect the secondary P6 and P7 anchor residues and would be expected to lead to differential binding activities. Finally, V in position 3 serves as a P1 residue for the DR1202 allotype only. In this binding mode, only D/E affects an HLA-binding residue while H/N and K/I are expected to point to the T-cell receptor. The p11 peptide elicits strong CD4 immunity and, remarkably, is commonly recognized across diverse human donors and HLA transgenic mice, related to its capacity to bind to multiple HLA-DR molecules. Blocking studies with the HLA-DQ monoclonal antibody, SPVL-3, suggest that a component of the response to p11 can be HLA-DQ restricted, which may further contribute to immunodominance. By contrast, the p18 peptide is a more promiscuous binder than the p11 peptide, although it is less frequently recognized by T cells. Several studies affirm that the number of memory antigen-specific T cells depends on the number of antigen-specific naive cells at the initiation of the response^34^–^36^ and in particularly, discriminate peptides with similar binding activity but differential T-cell-activating properties.^37^

SseB is one of many secretion system components that appear to feature strongly in the T-cell immunome of diverse bacterial infections including *Salmonella*, *Mycobacterium tuberculosis* and *Burkholderia*; perhaps the key example in this respect is ESAT-6 (EsxA), a part of the mycobacterial type VII secretion system.^38^

Although it is generally accepted that T cells play an important role in limiting *Salmonella* infections, the phenotype, specificity and location of these responses in humans remains unknown. The finding of strong SseB responses pre-existing in healthy controls, who have not had invasive *Salmonella* infections, suggests that these responses may be generated at the mucosa as a result of universal exposure to orthologous sequences derived from other pathogens and the bacteria of the human microbiota. This finding means that it is likely that such pre-existing responses are present among those who go on to develop typhoid after exposure, and that SseB immunity may therefore be insufficient to confer resistance to invasive *Salmonella* infections. On the basis of the current study and in the absence of CD45RA/RO flow cytometric assays, we also cannot formally exclude the possibility that a component of the very sizeable response to SseB may be contributed by priming of a response in naive cells. However, our observations are consistent with the role for SseB immunity shown in limiting infection during invasive salmonellosis as shown in the C57BL/6 protection studies with *Salmonella* SL134.^18^ Further studies are required to relate the magnitude of responses to attack rate in experimental challenge and duration and severity of infection.

## Supporting Information

**Figure S1.** Specific blocking of the response to p11 in donor EC02 by anti-HLA-DQ monoclonal antibody SPVL-3 added to cultures at 25 µg/ml.
