# Supplementary figures and images for: The serodominant secreted effector protein of Salmonella, SseB, is a strong CD4 antigen containing an immunodominant epitope presented by diverse HLA class II alleles
Source: Immunology. 2014 Oct 2;143(3):438–46. doi: 10.1111/imm.12327 (PMC4212957; doi:10.1111/imm.12327)

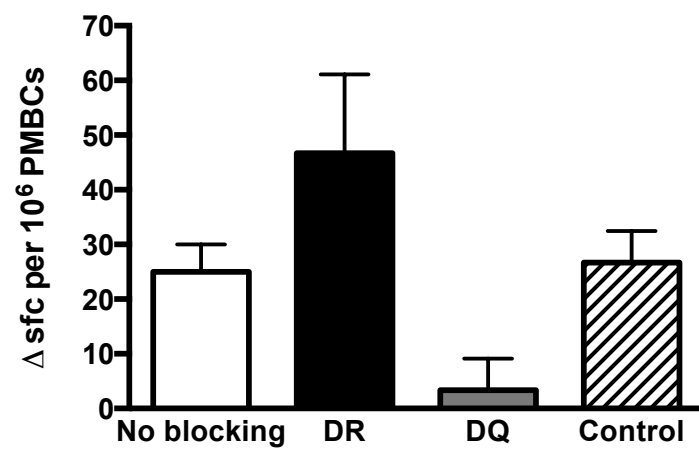

Supplement: Supplementary file 1 — Figure S1. Specific blocking of the response to p11 in donor EC02 by anti-HLA-DQ monoclonal antibody SPVL-3 added to cultures at 25 µg/ml. [file imm0143-0438-sd1.pdf]
